# A Community Engagement Process Identifies Environmental Priorities to Prevent Early Childhood Obesity: The Children’s Healthy Living (CHL) Program for Remote Underserved Populations in the US Affiliated Pacific Islands, Hawaii and Alaska

**DOI:** 10.1007/s10995-013-1353-3

**Published:** 2013-09-17

**Authors:** Marie Kainoa Fialkowski, Barbara DeBaryshe, Andrea Bersamin, Claudio Nigg, Rachael Leon Guerrero, Gena Rojas, Aufa’i Apulu Ropeti Areta, Agnes Vargo, Tayna Belyeu-Camacho, Rose Castro, Bret Luick, Rachel Novotny, the CHL Team

**Affiliations:** 1 University of Hawai‘i at Manoa, Honolulu, HI 96822 USA; 2 University of Alaska at Fairbanks, Fairbanks, AK USA; 3 University of Guam, Mangilao, GU USA; 4 American Samoa Community College, Mapusaga, AS USA; 5 Northern Marianas College, Saipan, MP USA

**Keywords:** Early childhood, Obesity prevention, Community-based, Pacific, Environment

## Abstract

Underserved minority populations in the US Affiliated Pacific Islands (USAPI), Hawaii, and Alaska display disproportionate rates of childhood obesity. The region’s unique circumstance should be taken into account when designing obesity prevention interventions. The purpose of this paper is to (a), describe the community engagement process (CEP) used by the Children’s Healthy Living (CHL) Program for remote underserved minority populations in the USAPI, Hawaii, and Alaska (b) report community-identified priorities for an environmental intervention addressing early childhood (ages 2–8 years) obesity, and (c) share lessons learned in the CEP. Four communities in each of five CHL jurisdictions (Alaska, American Samoa, Commonwealth of the Northern Mariana Islands, Guam, Hawai‘i) were selected to participate in the community-randomized matched-pair trial. Over 900 community members including parents, teachers, and community leaders participated in the CEP over a 14 month period. The CEP was used to identify environmental intervention priorities to address six behavioral outcomes: increasing fruit/vegetable consumption, water intake, physical activity and sleep; and decreasing screen time and intake of sugar sweetened beverages. Community members were engaged through Local Advisory Committees, key informant interviews and participatory community meetings. Community-identified priorities centered on policy development; role modeling; enhancing access to healthy food, clean water, and physical activity venues; and healthy living education. Through the CEP, CHL identified culturally appropriate priorities for intervention that were also consistent with the literature on effective obesity prevention practices. Results of the CEP will guide the CHL intervention design and implementation. The CHL CEP may serve as a model for other underserved minority island populations.

## Introduction

Childhood obesity prevalence and its associated health complications have become a major national and global public health issue. Obese and overweight children are at risk for serious chronic illnesses [1–16]. Striking disparity is found in US childhood obesity prevalence; indigenous groups, including US Affiliated Pacific Islanders, Native Hawaiians and Alaska Natives, are disproportionately affected [17–23]. For example, a state of emergency has been declared in the US Affiliated Pacific Islands (USAPI) due to the high prevalence of chronic health conditions in both adults and children in these island communities [24].

Individual level obesity prevention efforts promote short-term behavior change but may not have a significant or sustainable impact on childhood obesity [25] amidst an obesogenic environment [26]. Since the environment, i.e., the cumulative living conditions surrounding a child, is associated with childhood obesity [27–30], interventions for young children that use sustainable, multi-strategy, and multi-setting approaches are needed [31]. An ecological approach, which targets the individual, social and built environments, and policies [32, 33], expands the potential of prevention efforts to address critical upstream determinants of obesity-related behaviors, to influence larger populations and to have a long-term, sustainable impact [34]. Although evidence from ecological approaches is promising [35, 36], larger scale, adequately powered studies are needed.

A process for understanding which ecological approaches are most appropriate and have the highest probability of success over the long term is also needed. Young children are especially sensitive to environmental changes given their rapid growth and captive state as they are less able to exert personal choice within their family, school, and community environments [37]. Focusing on the environment as a mode for intervening to prevent early childhood obesity requires partnering with people that have first-hand knowledge of that environment (i.e., the people who live/experience that setting) to ensure applicability. In remote, underserved minority populations, such as within the USAPI, Hawaii and Alaska (will be referred to as USAPI/HI/AK throughout the remainder of the paper), a community-based approach to environmental interventions may be especially appropriate [38]. Community-based approaches allow for cultural context to be applied, trusting relationships to be forged and contributes to leveling the playing field between community members and researchers [39–42]. Community-based approaches focus on establishing relationships with community “experts” to build the community’s capacity to promote the desired outcome.

Community-based processes are also highly compatible with an assets-based philosophy, such as positive deviance, which is an approach to identify locally available, sustainable, and effective strategies suitable for a community [43, 44]. Positive deviance is based on the observation that in communities a few at-risk individuals follow uncommon, beneficial practices that result in better health outcomes than their neighbors who share similar risks [43]. Drawing on the local knowledge from these few individuals (i.e., positive deviants) to develop interventions has the potential to increase affordability, acceptability and sustainability of community-based action, since local culture is already well-integrated into the behaviors/practices that resulted in a positive outcome [43, 45].

Community-based approaches may be particularly critical for indigenous populations for whom mainstream models of childhood obesity prevention may have limited application. Through community partnerships cultural attunement is ensured leading to the strengthening of study design and implementation to more effectively address complex problems [41, 42, 46]. Community-based processes have been demonstrated to be a critical step to developing sustainable and successful young child obesity prevention programs for islands in the South Pacific [47, 48].

This paper describes the community engagement process (CEP) used by the Children’s Healthy Living (CHL) Program for remote underserved minority populations in the USAPI/HI/AK. CHL used the CEP to seek alignment and collaboration with community partners throughout Alaska, American Samoa, the Commonwealth of the Northern Mariana Islands (CNMI), Guam, and Hawai‘i to meaningfully address childhood obesity. This paper also highlights the overall priorities for environmental intervention strategies identified by communities for community-based, environmentally focused childhood obesity prevention in the USAPI/HI/AK Region and the lessons learned from the CEP. The information presented here can guide future children’s obesity prevention programs and policies and serve as a model for other island regions with remote, underserved native populations at high risk for obesity.

## Description of the Region and Program

### The US Affiliated Pacific Island (USAPI), Hawaii and Alaska Region (USAPI/HI/AK)

The USAPI/HI/AK region is vast and isolated, covering more area in the Pacific Ocean (one million square miles) than the contiguous US does on land. The ocean is viewed as part of the natural resource of the region, as land is on the American continent. The remote vastness of the region promotes a multitude of small, diverse, widely dispersed cultures (including subsistence cultures) living in unique environments with delicate ecosystems. Nonetheless, the USAPI/HI/AK is characterized by a number of shared strengths that can be leveraged to promote healthy living. Indigenous groups in the USAPI/HI/AK maintain a strong traditional culture that includes valuing the group in favor of the individual, respecting elders and the family unit, and prizing healthy subsistence foods (e.g., taro, fish). These attributes, coupled with the unifying US land grant college infrastructure throughout the region, present a unique opportunity to join together to create a larger voice to address childhood obesity and improve child health in the USAPI/HI/AK.

### The CHL Program

Collaborators from land grant colleges and universities [49, 50] in Alaska, American Samoa, CNMI, Guam, the Federated States of Micronesia (FSM), Hawai‘i, the Republic of the Marshall Islands (RMI), and the Republic of Palau (RP) formed the CHL Program. The land grant system [49, 50], one of the few unifying institutions across the USAPI/HI/AK, provided a suitable infrastructure for regional collaboration. The CHL Program was developed in response to the United States Department of Agriculture (USDA) Agriculture and Food Research Initiative to develop a multi-state/jurisdiction, multi-institutional, and multi-disciplinary team to integrate knowledge about child nutrition, physical activity, and social and environmental influences on childhood obesity in order to develop and implement a large-scale, multifaceted, community-based, and environmentally-focused intervention for preventing early childhood obesity (ages 2–8 years).

The overall goal of the CHL program is to strengthen the children’s environments to better promote active play and intake of healthy food in order to prevent early childhood obesity in the USAPI/HI/AK, which are located in the northern Pacific Ocean (except American Samoa). For CHL, the environment is broadly construed and includes the social, cultural, physical/built [51], political, and economic contexts of children’s lives. CHL seeks to develop, implement, and evaluate a community-based environmental intervention to address six target health behaviors proposed by the investigators and subsequently required by the funder (USDA): (a) increase the consumption of fruits and vegetables (b) increase water intake, (c) decrease intake of sugar-sweetened beverages, (d) increase physical activity, (e) increase the duration of sleep, and (f) decrease screen time (e.g., TV and recreational screen use).

## Methods: The CHL Community Engagement Process

**Fig. 1:**
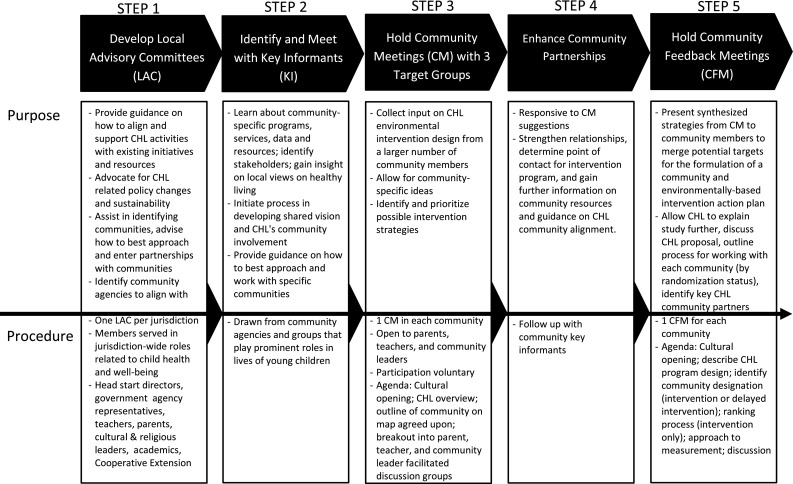
Children’s Healthy Living (CHL) Program community engagement process (CEP)

**Fig. 2:**
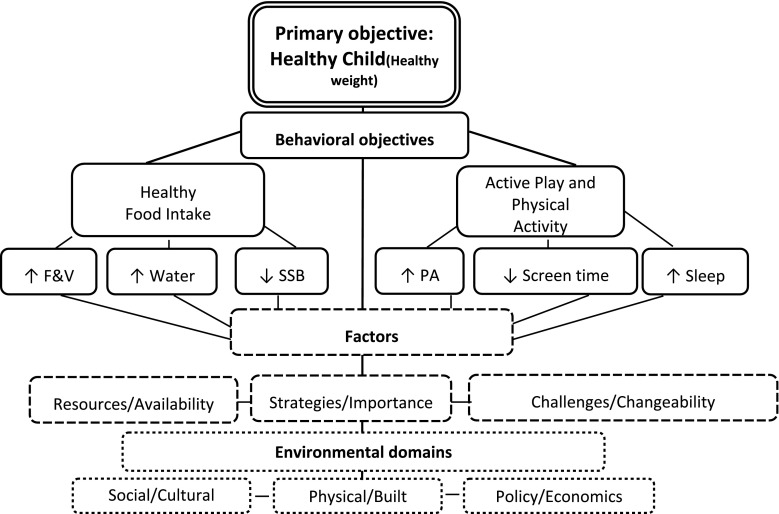
Children’s Healthy Living (CHL) Program conceptual framework for community engagement.* F&V* fruit & vegetable,* SSB* sugar-sweetened beverage,* PA* physical activity. The* double solid line boxes* represent the CHL primary objective of promoting a healthy child through a healthy weight. The* solid line boxes* relate to the six CHL target health behavioral objectives required by the funding agency. The* dash line boxes* relate to factors that influence the attainment of the CHL target health behavioral objectives: identifying resource types, availability and ease of access; possible intervention strategies prioritized by importance and feasibility; existing challenges to healthy behavior and the potential malleability of these obstacles. The* dotted line boxes* relate to the environmental domains that the factors that influence the attainment of the CHL target health behavioral objectives (see schematic: resources/availability, strategies/importance, and challenges/chanegability) operate in.

The overall goal of the CHL CEP was to foster partnerships with CHL communities to jointly develop a community-based, multi-level, sustainable environmental intervention to prevent childhood obesity. The CHL CEP was informed by the analysis grid for elements linked to obesity (ANGELO) action model, a community and ecologically based framework used to develop environmental interventions to reduce childhood obesity in three island nations and a community in Australia, located in the South Pacific region [31, 37, 52, 53]. The ANGELO action model includes both a conceptual framework for analyzing obesigenic environments and a process model for engaging community stakeholders. In the ANGELO conceptual framework, environments are cross-categorized by size and type [37]. In the ANGELO process model, community members and researchers use the conceptual framework to analyze the assets and liabilities of a community’s environment, prioritize areas amendable to productive change, and develop an action plan [37]. Therefore, the CHL CEP was a multi-step process guided by a CHL specific conceptual model that engaged key stakeholders through a local advisory committee, key informant interviews, community meetings (CM) and community feedback meetings (CFM) (see Fig. 1 for a description of the purpose, membership, and process of each group and Fig. 2 for the CHL conceptual framework for community engagement). In this paper we focus on the CM and CFM, the goals of which were to (a) identify each CHL community’s assets and needs relating to healthy eating and active living, and (b) prioritize environmental intervention strategies relating to healthy eating and active living in order to inform intervention development.

Institutional Review Board approval from the University of Alaska Fairbanks, University of Guam, and University of Hawai‘i at Manoa were attained prior to the initiation of the CEP. American Samoa Community College and the Northern Mariana College ceded their Institutional Review to the University of Hawai‘i at Manoa.

In addition, approvals for working with PreSchool and Head Start (a US federally funded program that educates preschool-age children and their families) teachers and parents were received in coordination with the program directors and/or boards (e.g., Tribal), when appropriate, in all jurisdictions. Other local level approvals included approvals from the chiefs (matai) and ministers (faifeau) of pertinent American Samoan villages and the participating village mayors in Guam.

### Selection of Target Communities within Each Jurisdiction

Four communities were selected in each of the five jurisdictions in order to form two matched pairs per jurisdiction for a total of 20 communities. Later, each community with a matched-pair was randomized to intervention or delayed intervention. Community selection was based on the following eligibility criteria, identified using the 2000 US Census tract data [54]: population size of >1,000, >25 % of the population of indigenous/native descent (15 % in Alaska due to no census tract with population of 1,000 having more than 25 %), and >10 % of the population under age 10 years (based on combining census tract data groups of <5 years of age and 5–9 years of age, in order to have sufficient population for CHL targeting of 2–8 year olds). Additional criteria included adequate settings for sampling children (e.g., schools), that children live and go to school in the same community, minimal risk of contamination between matched-pair communities, reasonable accessibility for the CHL team, community cohesiveness, and sufficient settings for intervention (e.g., community centers, parks, churches, and stores).

### Recruitment Methods

A non-probability, convenience sampling scheme was used to recruit participants for the CM and CFM from each of the selected CHL communities (see Fig. 1). Participants that either resided or worked in the target communities were invited to attend. Each jurisdiction used their Local Advisory Committees (LAC) and key informants to assist with developing lists of potential participants who would be good representatives of one of three constituent groups—community leaders, teachers, and parents. Additional potential participants were identified using contact and member listings from consortia and organizations sharing similar objectives (e.g., Non-Communicable Disease Consortium) or serving similar populations (e.g., early childhood education centers) as CHL and CHL staff contacts within the communities.

The recruitment process in each jurisdiction followed specific cultural protocols. In Hawai‘i, Guam, and Alaska, CM and CFM recruitment flyers were distributed over professional networks or paper copies were posted at various locations in the community. Letters of invitation were also hand delivered and emailed. In CNMI, the CHL team recruited participants via email and telephone. In American Samoa, the recruitment method used an involved and protracted cultural protocol under the direction of a High Orator Chief (Author AARA) who met one on one with each potential participant based on a chain of contact system.

### Community Meetings (CM) and Community Feedback Meetings (CFM)

The goals of the CMs and CFMs were to identify assets and needs around healthy eating and active living among children ages 2–8 years and to identify priorities for the intervention in CHL communities. Specifically, facilitated group discussions: (a) identified factors that promote or hinder healthy living, (b) identified community resources that could be leveraged to promote healthy living, and (c) prioritized potential environmental intervention strategies.

Questions for the CM facilitated group discussions were developed in accordance with the CHL conceptual framework (see Fig. 2) and the positive deviance approach (e.g., focusing on the strengths) [43]. Separate questions were written for each of the three constituent groups—parents, teachers, and community leaders—to elicit constituent-specific ideas (see Table 5 in “Appendix”). For example, teachers were asked whether their school allowed sugar sweetened beverages, while community leaders were asked about local policies related to sugar-sweetened beverages. In instances when participants identified with more than one constituent group (e.g., parent and teacher), they were asked to select and participate in the group they most strongly identify with. Questions were pre-tested to ensure cultural appropriateness and clarity. The CFM were held after the CM and the facilitated group discussions focused on the assets, needs and resources identified in the CM.

The CMs and CFMs were guided by trained facilitators who were instructed to remain neutral to the discussion [55]. Ground rules were agreed upon prior to the start of every meeting to ensure a safe and open venue for communication [55]. CM and CFM discussions were recorded on flip chart paper. The written record served as the group memory and was used to facilitate CM and CFM discussion [55]. Constituent group discussions were also recorded using digital recorders to provide further detail during analysis. Meetings were conducted in English in all jurisdictions except in American Samoa where Samoan was used.

### Qualitative Analysis—Community Meetings (CM)

The group memory served as the primary tool for qualitative analysis for the CM. Participants were asked to prioritize key points elicited during the CM based on importance and changeability. Prioritization was determined by a facilitated group agreement process in which before moving on to the next discussion item participants were asked to confirm if they could live with and agree to the prioritized items [55]. In Guam and in one of the constituent group (parents) in Hawai‘i, prioritization of the CM group memory was achieved by thematic content analysis through a clustering/coding process [56] using transcribed group memory responses to yield a list of the three to five main priorities made in response to each question. The priorities identified in the group memories were then aggregated across the parent, teacher, and leader groups in each community to form community-specific priorities. Because questions for each group were similar but not identical, questions and responses were clustered by topic or content area. Data from the four communities per jurisdiction were then aggregated to identify jurisdiction priorities. Priorities from each jurisdiction were then compared to identify CHL-wide themes.

### Qualitative Analysis—Community Feedback Meetings (CFM)

For the CFM, analysis was begun at each jurisdiction. For the purposes of intervention development, only the communities randomized to intervention were included in the analysis. The CFM in the delayed intervention communities focused on the CHL delayed intervention proposal and timeline. Jurisdiction- and community-specific priorities for environmental intervention strategies to promote each target behavior were shared. A facilitated discussion regarding the proposed environmental intervention strategies was held [55] and then participants ranked in each CHL target health behavior their top two (based on importance and changeability) proposed environmental intervention strategies. Another facilitated discussion followed to gather further feedback on the ranking process. After the meeting, voting results were tabulated for each CHL target health behavior for each state/jurisdiction. Jurisdiction voting results were then combined to identify the most endorsed CHL wide priorities for environmental intervention strategies.

## Results

The CHL CEP was implemented over a 14 month period (April 2011–June 2012). In this time period, each CHL jurisdiction met with their LAC at least twice. CM were conducted between November 2011 and February 2012 after the initial LAC meetings and multiple community key informant meetings. CFM were held between May and June 2012. Across the 5 jurisdictions 912 individuals representing a range of stakeholders participated in the CHL CEP (See Table 1). Parents and teachers were especially well represented in the CHL CEP. In many instances, participants who attended the CM also attended the CFM.

**Table 1:** Community representatives for Local Advisory Committees (LAC), key informants (KI), community meetings (CM), and community feedback meetings (CFM) across all Children’s Healthy Living (CHL) Program jurisdictions

Community representatives	LAC	KI	CM	CFM	TOTAL
Education					
Head Start*	4	22	78	6	110
Preschool	1	15	35	7	58
Department of Education	4	14	18	5	41
Other^‡^	13	35	41	26	115
Health Services	11	32	23	21	87
Social Services	0	10	7	3	20
Government^§^	15	27	32	32	106
Food Supply	2	19	5	1	27
Wellness^†^	1	9	9	3	22
Other**	8	39	34	58	139
Parents	3	28	127	29	187
Total	62	250	409	191	912

* US federally funded program that educates preschool-age children and their families

^‡^College, childcare centers/daycares, elementary schools, unspecified education type

^§^Supplemental Program for Women, infants and children (WIC), parks and recreation, chiefs, mayors, cooperative extension service, affairs office, Department of Health

^†^Sports groups, gyms, health advocates

** Church, businesses, associations, unspecified community representatives

Community meeting priorities for environmental intervention strategies that were identified in all CMs held in a CHL state/jurisdiction are presented in Table 2. The four communities in American Samoa shared the highest number of priorities while the four communities in CNMI shared the least. The priorities for environmental intervention strategies that were most commonly suggested across CHL are identified in Table 3. Access to healthy, locally-grown food was a priority common across all five jurisdictions. Influencing policies (both school and governmental) to incur healthier behaviors were also identified as important and changeable in four out of five jurisdictions. Limiting screen time was a priority only in American Samoa and Hawai‘i. All participants received a brief summary of community-specific meeting findings in formal letters. CM participants were in support of CHL working in partnership with their communities to develop the CHL Program.

**Table 2:** Children’s Healthy Living (CHL) Program priorities for environmental intervention strategies identified in all community meetings held in each corresponding jurisdiction

CHL Jurisdiction				
Alaska	American Samoa	CNMI	Guam	Hawaii
Priorities for environmental intervention strategies				
1. Value system emphasizing self-reliance (e.g., subsistence lifestyle) combined with a sense of community an asset for healthy living	1. American Samoa Community College Community and Natural Resources should lead the dissemination of healthy eating and physical activity information for the community	1. Nutrition Assistance Program (NAP) should mimic Supplemental Program for Women, Infants, and Children (WIC) to restrict food purchases of unhealthy food and drinks	1. Activities should be focus on the broad-spectrum of the community involving adults that influence young children (e.g., parents, teachers, caregivers)	1. Strategies locally and culturally based (e.g., incorporate concepts like makahiki, ahupua‘a, ohana, hula)
2. Family education on all aspects of healthy living	2. Family plantations are important to increasing fruit and vegetable intake		2. Improve physical activity infrastructure development, maintenance and access	2. Older siblings/children as healthy role models
3. Increase awareness and access to the diversity of resources for healthy living	3. Adequate water resources (e.g., water coolers) should be readily available so children can only be given water to drink			3. Give families specific activities to replace screen time
	4. Importance of role models demonstrating healthy living			

CNMI = Commonwealth of the Northern Mariana Islands, Makahiki = traditional Hawaiian festival, Ahupua‘a = traditional Hawaiian land division usually extending from the uplands to the sea, Ohana = family in Hawaiian, Hula = traditional Hawaiian dance

**Table 3:** The most commonly suggested priorities for environmental intervention strategies identified in all Children’s Healthy Living (CHL) program community meetings

Overall CHL priorities for environmental intervention strategies	Alaska	American Samoa	CNMI	Guam	Hawaii
1. Educate parents, siblings, grandparents, children, communities on healthy living	X	X	X	X	X
2. Better and more free community activities and resources to promote healthy living	X		X	X	X
3. Importance of family, teachers, leaders, other respected figures as role models setting a healthy living example		X	X	X	X
4. Improve drinking water access/facilities		X	X	X	X
5. Community resources maintained and accessible during all times making physical activity easier	X		X	X	X
6. School policies need to be changed to make school lunches healthier, encourage water intake, increase physical activity, and reduce sugar sweetened beverage			X	X	X
7. Limit screen time		X			X
8. Change government policies to promote healthy lifestyle, regulate use of government assistance		X	X	X	X
9. Healthy locally-grown food, easily accessible and affordable	X	X	X	X	X

*CNMI* Commonwealth of the Northern Mariana Islands

Environmental intervention priorities focused on infrastructure, access, role modeling and education were the most endorsed (e.g., received the most votes) across the CHL region for the CHL target health behaviors (See Table 4). Feedback from community members during the CFM-facilitated discussion stressed that to ensure CHL program success and sustainability, communities need to take ownership of the CHL initiative (Guam and American Samoa) and that CHL needs to incorporate cultural practices (CNMI, Hawai‘i and American Samoa) and be a catalyst for enhancing local resources/programs already directed at tackling the childhood obesity issue (Alaska and Guam).

**Table 4:** Children’s Healthy Living (CHL) program priorities for environmental intervention development endorsed by community members at community feedback meetings held across the CHL region to affect CHL target food and activity behaviors

Children’s Healthy Living target food and activity behaviors					
Increase sleep	Increase PA	Decrease screen time	Increase F/V	Increase water	Decrease SSB
Priorities for environmental intervention development					
1. Healthy lifestyle education	1. Provide affordable/free community resources and programs	1. Provide/promote alternative, community, and sports activities	1. Teach family and children about healthy living to promote F/V intake and role modeling	1. Allow only water at events (e.g. church, school, birthdays, sports activities)	1. Teach family and children about beverage options and benefits of water
2. Regular sleep times	2. Organized activities and gear/equipment lending program for children and families	2. Educate parents	2. Promote home/community gardening through school and community gardening education	2. Healthy lifestyle education to teach family and children about healthy beverage options and benefits of water	2. Promote healthy nutrition
3. Physical activity schedules	3. Animal control	3. Build better infrastructure for alternative activities	3. More F/V in school meals	3. Increase access to clean water in school and public places	3. Healthy lifestyle education
	4. Build and maintain indoor/outdoor infrastructure	4. Parents monitor children’s screen time			4. Limit access and consumption of through government assistance programs

*PA* physical activity, *F*/*V* fruit/vegetable, *SSB* sugar-sweetened beverages

## Discussion

The multi-step CEP successfully prioritized environmental intervention strategies for intervention program development in participating communities from the USAPI/HI/AK. These priorities focused on policy development, enhancing access to locally grown fruit and vegetables, engaging identified role models (e.g., parents, grandparents, older siblings), increasing access for safe physical activity venues and to clean water, and the provision of education to young children ages 2–8 years and to other influential adults to support healthy eating and physical activity. The priorities span the multiple levels of influence on a child’s health, ranging from the individual level (e.g., dietary intake) to the governmental level (e.g., policy) suggesting that following an ecological, community-based approach such as the ANGELO model is a viable approach [52]. Since recommendations have been made in the literature [57] to encourage the publishing of formative research on program development, and because the USAPI/HI/AK populations are underrepresented in the literature, a major objective of this paper was to provide to the wider scientific audience the CHL CEP used in the underserved and minority populations of the USAPI/HI/AK.

Interestingly, many of the environmental intervention strategies are similar to other previously successful childhood obesity prevention approaches [47, 58–65]. Group agreement and participant voting was the primary method used for the prioritization to ensure that identified priorities were community driven [55], while aligning with program behavioral objectives. No information on evidence-based priorities was provided to participants prior to the CM or the CFM. The alignment between the evidence-base and the community’s perspective suggests that the community is an appropriate resource to determine how CHL can positively affect the environment to promote healthy eating and physical activity, a finding well received by community members at the end of the CEP.

The ability to identify community priorities for environmental intervention strategies may have been an outcome of bridging the gap between constituencies. Community leaders, parents, and other members of the community invested in child health were invited to the table to share openly and honestly. For example, at the onset of all CM and CFM, CHL staff and participants agreed upon ground rules to create a safe space for discussion [55]. Impartial facilitators promoted full participation ensuring that all had an opportunity to share while the use of facilitated small group discussions among like constituencies allowed for a less intimidating environment. We found that the communities were grateful for the opportunity to discuss the threat of childhood obesity in their community and were eager to provide input. Overall, participants assumed community responsibility and recognized the importance of all levels of the community working together to address the problem.

The CEP allowed CHL to identify priorities for environmental intervention strategies for the USAPI/HI/AK so that the CHL team could build a sustainable intervention operation framework for implementation. However, since no community is alike, especially spanning the Pacific Rim, applying the findings into developing each community’s intervention program is the next step. A positive aspect of the CHL CEP was that it allowed for the unique priorities, assets, and resources of each participating community to be identified. Correspondingly, the process assisted in the identification of potential community champions, especially evident by community partners who were involved in multiple stages of the CEP, who are significant players in community-based intervention success [52]. The CEP also ensured that a jurisdiction’s unique issues could also be identified. For example, in American Samoa and the CNMI, the cultural preference for oral versus written communication of messages was expressed multiple times. Oral interaction was identified as important to ensure that the interpretation of results (e.g., priorities for environmental intervention strategies) is culturally-based. These findings will influence the dissemination strategies for intervention messages in American Samoa and CNMI. None-the-less, there was also motivation to be recognized as a USAPI/HI/AK Regional group, recognizing the potential additional power of a shared regional vision in affecting policy and other change.

One limitation to our community-based processes related to balancing the intentions of a community-based and participatory process with our requirements and obligations as a federal grantee. The CHL team recognizes that a truly community-based participatory research (CBPR) process [66] was not used. Though the intent was to use CBPR, many of the parameters and structure of the program were required to be set prior to receipt of the grant. As a result, community members did not have complete leeway to set project goals, objectives, and outcomes. For example, the six outcomes and target behaviors to prevent childhood obesity were set during the initial grant application process (although they built on prior work with the USAPI and Hawaii communities). Also, meeting the scientific expectation of a level of standardization of methodological approach across the culturally diverse USAPI/HI/AK where access to resources (e.g., high speed internet) is quite varied is challenging. Grant writing, where structural domains are set in place, does not lend itself to truly being CBPR [67] as it lacks the flexibility inherent to the CBPR process [65]. However, the CHL Program is an outcome of many years of collaborative, community-based work among partners in the USAPI and Hawaii [20, 68–72]. The pre-planning for CHL included a grant application regional planning meeting (May 2011), which involved a variety of stakeholders and professionals in the field from the USAPI/HI/AK, who all agreed upon the strongest leadership profile to ensure successful competition for the grant. A grant of this scale continues to pose the management challenge of balancing between research structures and being community-based. With many existing partners, bringing new partners into the management structure is challenging, so compromise and adherence to common protocols is needed to yield group results. In most cases, the USAPI/HI/AK’s cultural value of the group and the power of contributing to something larger than one’s own community facilitates CHL’s progress.

One important outcome of the CHL CEP was the lessons learned. As mentioned earlier, the USAPI is especially resource-limited reinforcing the need to collaborate with not only land-grant institutions and governmental agencies (e.g., Department of Health) but also with community-based organizations and agencies that have a vested interest in health and who are ultimately going to sustain health in the local communities. Establishing community liaisons (e.g., community champions) became essential to ensure appropriate linkages with the agencies and organizations that span the USAPI/HI/AK were formed. Forming these linkages required an intensive investment of time to ensure that appropriate cultural protocols were followed, especially for US and Non-US Affiliated Pacific Islander populations who prefer oral and group processes [73]. Though challenging in research protocols, being adaptable and flexible is also essential. For example, CHL staff needed to quickly adapt to situations in community meeting events when not everyone that is invited attends or when attending individuals were not originally invited. Jurisdictions also had to be willing to reschedule activities that conflicted with the holiday season or other cultural events. Another important aspect is that what works in one jurisdiction does not always work in another (e.g., comment cards were not considered appropriate in American Samoa). Rather than developing rigid protocols for implementation, guideline templates are put forth so each jurisdiction can identify and discuss with the coordinating work group how to make adaptations, if needed.

As demonstrated in the OPIC Study in the South Pacific [47], engaging the community in the intervention development process significantly impacts CHL intervention effectiveness [37, 52]. The CEP ensured that the communities not only provided the initial input but also prioritized and verified that community interpretations resonated and were culturally appropriate. For example, the CEP provided specific language, examples, and the culturally contextualized perspective. Repeated engagement allowed for community validation, which is important in collectivist cultures of the USAPI, Hawaii and the Native populations of Alaska, and will be influential during intervention implementation.

## Conclusion

The CHL CEP was a viable community-based process covering a vast region with a variety of cultures. It allowed for flexibility while integrating commonalities. The CHL CEP identified community-based priorities for environmental intervention strategies that would inform CHL intervention program development and implementation in Alaska, American Samoa, CNMI, Guam and Hawai‘i. These priorities focused on promoting healthy eating and physical activity policy, training and supporting role models, enhancing access to fruits, vegetables, water and safe play and providing education/training. The CHL CEP is being adapted for use in the FSM, RMI, and RP. The approach taken by CHL to develop a community-based environmentally-focused child obesity prevention intervention may also be useful for other regions of the Pacific or in other underserved, minority island populations.

## Appendix

See Table 5.

**Table 5:** Children’s Healthy Living (CHL) program community meeting discussion questions by constituent group (e.g., parents, teachers, and community leaders)

Parents		
1	What does a healthy young child look like to you?	
	Follow-up	What keeps a young child healthy?
2	Where are fruits and vegetables available in your community?	
3	What helps young children to eat more fruit and vegetables in your community?	
	Follow-up	What makes it hard for children in the community to eat fruits and vegetables?
	Follow-up	What can you do to help young children eat more fruits and vegetables, at home? At school?
	Follow-up	What can we do to increase young children eating more fruits and vegetables at home? At school?
4	What are some of the reasons that less healthy foods are chosen over the healthy foods in your community?	
	Follow-up	What kinds of healthy foods, like fruits and vegetables, are available in your community that could be eaten instead of the less healthy foods like chips, cookies, and candies?
5	What can we do in your community to encourage young children to drink more water?	
	Follow-up	What can we do to help young children to drink less sugar-sweetened beverages like in your community?
6	At what time of day, or when are young children more likely to be active?	
	Follow-up	Where are young children likely to be more active?
7	What opportunities are there for young children to be physically active, like to walk or be involved in active play – running around, sports, etc. in your community?	
	Follow-up	What makes it hard for young children to engage in active play?
8	How do you see TV, computers, computer games influencing your young child’s activity?	
	Follow-up	When does watching TV, computers and computer games influence your young child’s activity, during the day or night?
	Follow-up	Does it affect their sleep?
9	Do you limit their time watching TV, video games, or using the computer? How do you do this?	
10	What changes to your community or your surroundings would you recommend to decrease screen time and increase active play?	
	Follow-up	Besides TV, computers and computer games what other activities are available for young children?
11	How does your culture shape the food that your young child eats? How does your culture shape your young child’s active play?	
	Follow-up	What traditions/cultural practices do you use at home to promote healthy eating? Active play?
	Follow-up	What traditions and cultural practices can encourage healthy eating? Active play?
	Follow-up	What traditions/cultural practices make it difficult for young children to eat healthy? To be physically active?
12	How important is healthy living (eating healthy and being active) to other family members?	
13	What positive environmental changes in the community would help parents and young children to eat healthy? Be more active?	
	Follow-up	What would be an ideal community program that would increase your child’s physical activity? Improve his/her eating habits?
	Follow-up	Can you share some ways that you have been successful in getting your children to eat healthy? Be active?

Teachers		
1	What words would you use to describe a healthy young child at your school/program?	
2	Describe how the food choices of the young children in your school/program affect their health right now?	
	Follow-up	Describe how the current eating habits of young children in your school/program will affect their health in the future?
3	What changes can be made in your school/program to get young children to:	
	a. Eat more fruits and vegetables?	
	b. Drink more water?	
	c. Get children to drink less sugar-sweetened beverages?	
4	What kind of healthy foods, can your school/program offer to young children to replace the less healthy ones like chips, cookies, and candies?	
5	What are the sources of healthy food in your school/program that are available to young children?	
	Follow-up	What makes it difficult to use these sources?
	Follow-up	How can these sources be strengthened or encouraged?
6	What makes it easy for young children at your school/program to be physically active?	
	Follow-up	How can we strengthen or encourage?
7	What are the places or organizations located near your school/program that young children and their families go to be active?	
	Follow-up	What makes it easy to use these resources?
	Follow-up	What makes it difficult to use these resources?
8	Do young children in your school/program come well rested?	
	Follow-up	For those children who are not well rested, how might caregivers be encouraged to help young children be more well rested?
9	What could you recommend to lower young children’s screen viewing time, or time spent in front of the TV, computers, computer games, in order to allow more opportunity for other activities such as active play?	
10	What needs to be done to promote healthy living (eating healthy and being active) for young children in your community?	
	Follow-up	What do you think would be the ideal strategy to increase active play in your school/program? Healthy eating?
11	What more can your school/program do to increase healthy food availability in your community? Physical activity?	

Community leaders		
1	How does your community promote healthy eating and physical activity for young children?	
	Follow-up	What are some examples of community activities or strategies that promote health?
	Follow-up	What would you like to see in your community to improve and/or promote healthy eating and physical activity?
2	What can you do or change in your community to help young children eat more healthfully?	
	Follow-up	Where are fruits and vegetables available in your community?
	Follow-up	What are ways (e.g., resources) to increase fruit and vegetable availability in your community?
	Follow-up	What are ways (e.g., resources) to increase fruit and vegetable consumption in your community?
	Follow-up	We recognize that it may be hard but what has made it easier for your community to increase the availability and consumption of fruits and vegetables?
3	What community changes or other community based approaches can you think of that helps promote young children to drink more water?	
	Follow-up	What policies or programs can your community promote or do to discourage consumption of sugar-sweetened beverages?
	Follow-up	How can we increase resources currently available in your community to promote water intake?
4	Who are the people or organizations in your community that promote healthy eating?	
	Follow-up	What are the things your community is doing that promotes healthy eating?
	Follow-up	What are other sources of information for families in your community that promote healthy eating?
5	What existing programs, infrastructure, policies or other resources support young children to be physically active in your community?	
	Follow-up	Please discuss how well these resources are used by the community?
	Follow-up	*If response is none or very little, ask* what are the some of the reasons they are not used?
	Follow-up	What do you believe can be done to improve/increase physical activity for young children in the community?
6	If you wanted the children in your community to spend less time in front of the television, computer and video game screen, what changes to your community or your surroundings would you recommend?	
	Follow-up	If necessary, what makes it easy for young children to spend time in front of the screen?
7	Who are the people or organizations in your community which promote physical activity for young children?	
	Follow-up	What are the things your community is doing to promote physical activity for young children?
8	What are the best ways to get information on healthy eating and being active to families in your community?	
	Follow-up	What can we do to make children eating healthier and being physically active a priority in your community?
9	Thinking outside of the box, what are some ideas, policies or model programs that you have or can think of that would enable your community to enhance young children eating healthy and being active?	
